# Candidate Gene for Kernel-Related Traits in Maize Revealed by a Combination of GWAS and Meta-QTL Analyses

**DOI:** 10.3390/plants14060959

**Published:** 2025-03-19

**Authors:** Hanlong Dong, Zelong Zhuang, Jianwen Bian, Rui Tang, Zhenping Ren, Yunling Peng

**Affiliations:** 1 College of Agronomy, Gansu Agricultural University, Lanzhou 730070, China; hdl15888@163.com (H.D.); zhuangzl3314@163.com (Z.Z.); bjwen1018@163.com (J.B.); tangr7450@163.com (R.T.); renzp1003@163.com (Z.R.); 2 Gansu Provincial Key Laboratory of Aridland Crop Science, Gansu Agricultural University, Lanzhou 730070, China; 3 Gansu Key Laboratory of Crop Improvement & Germplasm Enhancement, Gansu Agricultural University, Lanzhou 730070, China

**Keywords:** maize, kernel traits, GWAS, meta-QTL, candidate gene

## Abstract

Maize kernel traits represent crucial agronomic characteristics that significantly determine yield potential. Analyzing the genetic basis of these traits is essential for yield improvement. In this study, we utilized 1283 maize inbred lines to investigate three kernel-related characteristics: kernel length (KL), kernel width (KW), and 100-kernel weight (HKW). We conducted a genome-wide association study (GWAS) on three kernel-related traits, resulting in the identification of 29 significantly associated SNPs and six candidate genes. Additionally, we compiled quantitative trait loci (QTL) information for 765 maize kernel-related traits from 56 studies, conducted a meta-analysis of QTL, and identified 65 meta-QTLs (MQTLs). Among the 23 MQTLs, we found 25 functional genes and reported candidate genes related to kernel traits. We identified 26 maize homologs across 19 MQTLs by utilizing 25 genes that affect rice grain traits. We compared the 29 significant SNPs detected with the physical locations of 65 MQTLs and found that 3 significant SNPs were located within these MQTL intervals, and another 10 significant SNPs were in proximity to these intervals, being less than 2 Mb away, although they were not included within the MQTL intervals. The results of this study provide a theoretical foundation for elucidating the genetic basis of maize kernel-related traits and advancing molecular marker-assisted breeding selection.

## 1. Introduction

Maize, widely recognized as the “golden crop”, is essential for the sustainable development of animal husbandry and industry, playing an irreplaceable role in maintaining global food security and stability [1,2]. Increasing maize yields has long been a primary objective for breeders [3]. Key kernel properties, such as kernel length (KL), kernel width (KW), and 100-kernel weight (HKW), directly influence maize yield [4]. Consequently, investigating the genetic mechanisms underlying maize kernel traits is crucial for enhancing maize yield and informing breeding practices.

Maize kernel-related traits, which are crucial for yield, are highly heritable quantitative traits that are less influenced by environmental factors, and their expression is governed by micro-effect polygenes [5,6,7]. In recent years, numerous scholars, both domestically and internationally, have conducted genome-wide association studies (GWAS) and quantitative trait locus (QTL) analyses focused on maize kernel-related traits, resulting in the identification of a significant number of SNPs and QTLs. Qu et al. [8] utilized the fixed and random model circulating probability unification (FarmCPU) of GWAS to analyze the kernel traits such as kernel length, kernel width, and kernel thickness of 212 excellent maize inbred line materials and detected 47 significant SNPs and 58 candidate genes on 10 chromosomes of maize. Xiao et al. [9] employed the generalized linear model (GLM) of GWAS to analyze the kernel volume and kernel weight of 209 sweet maize inbred lines. They identified a total of 15 significant SNPs associated with either kernel volume or kernel weight and subsequently mined 15 candidate genes. Veldboom et al. [10] constructed an F_2:3_ population comprising 150 maize inbred lines derived from Mo17 and H99. They conducted QTL localization of kernel-related traits and identified five QTLs associated with KL and six QTLs related to HKW. Zhu [11] utilized the DH population as the foundational population for the identification of target trait locations. By integrating this approach with the complete interval mapping method, six QTLs associated with kernel length were identified. Tang et al. [12] employed the composite interval mapping method to conduct QTL positioning in the “Immortalized F_2_ Population” (IF_2_) of maize, successfully identifying five QTLs associated with HKW. Li [13] utilized maize inbred lines KA105 and KB024 to construct one recombinant inbred line (RIL) population and two immortalized backcross (IB) populations for the parental lines. Through QTL mapping, 38 QTLs associated with kernel traits were identified. He et al. [14] used the F2:3 family lines constructed from the tropical maize inbred lines T32 and QR273 as materials and combining the evaluation results of grain-related traits in two different environments, a total of 52 QTLs controlling maize grain-related traits were identified using the complete interval mapping method, including 3 major QTLs. Although a large number of QTLs have been mined at present, due to differences in experimental environment, mapping methods, population size, population type, trait selection, and statistical methods, the localization QTL confidence intervals are relatively scattered, and the location results are relatively scattered. It is difficult to form a unified conclusion, making it very few QTLs effectively applied to breeding practices, such as marker-assisted selection [15]. However, a meta-QTL analysis can aggregate these dispersed results, leading to a reduction in the QTL confidence interval. This enhancement improves the accuracy of QTL localization, minimizes false positive results, and uncovers more reliable genetic markers, which are of great significance for crop improvement and functional gene mining [16].

Currently, meta-QTL analysis is extensively utilized in crop genetic breeding. Liu et al. [17] conducted a meta-analysis of 381 QTLs associated with wheat yield, identifying 86 meta-QTLs (MQTLs) related to yield and uncovering 210 candidate genes. Anilkumar et al. [18] compiled rice grain weight QTL information from various groups and environments, conducted a meta-QTL analysis, and identified three significant grain weight MQTLs on chromosome 3, as well as five MQTLs associated with early grain development. Truntzler et al. [19] conducted a meta-analysis using the results from 11 different mapping experiments to identify QTLs associated with plant digestibility and cell wall composition in maize. They identified 68 MQTLs related to the quality of silage maize. Tang et al. [20] used 697 initial QTLs related to maize quality traits for meta-analysis, identified 41 MQTLs, and mined nine candidate genes related to maize quality traits in combination with GWAS.

The increase in corn yield is an extremely complex process, and we have chosen to focus on the kernel-related traits that have the most direct impact on it. Although previous studies have identified multiple SNPs or QTLs associated with maize kernel traits through GWAS or QTL mapping, the synergistic effects of combining meta-QTL and GWAS for the analysis of maize kernel-related traits remain unclear. Moreover, to date, there have been relatively few studies that integrate GWAS and meta-QTL analyses to dissect the genetic underpinnings of maize kernel-related traits. Therefore, We will employ multi-level genetic analysis methods, combined with large-scale samples and cross-species comparisons, to systematically elucidate the genetic basis of maize kernel traits. We utilized the CUBIC [21] population of 1283 maize inbred lines for GWAS analysis of three maize kernel-related traits. In addition, we integrated QTL information for kernel-related traits in different maize populations and identified “consistent” QTL related to maize kernel traits through meta-analysis, which was then validated against the GWAS results of the optimal model to identify potential candidate genes. We hope that our results will establish a foundation for analyzing the genetic mechanisms underlying maize kernel size, thereby providing both a theoretical basis and technical support for enhancing maize yield and facilitating molecular marker-assisted selection in breeding.

## 2. Results

### 2.1. Phenotypic Analysis of Kernel-Related Traits

Statistical analysis of phenotypic variation demonstrated that all measured maize kernel-related traits displayed continuous variation patterns, with kernel length (KL), kernel width (KW), and 100-kernel weight (HKW) showing coefficients of variation of 8%, 8%, and 18%, respectively (Table 1). All traits followed a normal distribution, as illustrated in Supplementary Figure S1, indicating that these kernel-related traits are typical quantitative traits, and their underlying genetic mechanisms are primarily governed by multiple genes.

Correlation analysis (Supplementary Table S1) revealed that each trait exhibited a highly significant positive correlation. This finding suggests that the traits associated with maize kernels interact and mutually influence one another, collectively regulating the size of the kernels. In summary, the three kernel-related traits examined in this study are well-suited for GWAS.

### 2.2. GWAS Analysis

**Figure 1:**
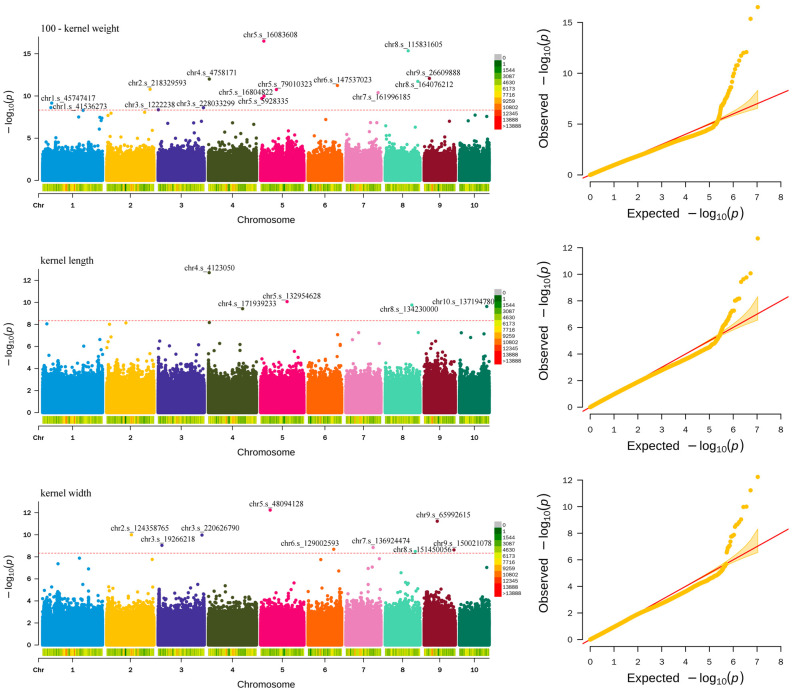
Manhattan diagram and QQ diagram of maize kernel-related traits under FarmCPU model analysis. The red dotted line represents the threshold line.

To identify the optimal model for GWAS concerning grain traits associated with corn yield, we conducted GWAS analyses for each trait utilizing both the generalized linear model (GLM) and the fixed and random model circulating probability unification (FarmCPU). The results indicate that the FarmCPU model effectively manages both false positive and false negative outcomes in association analysis, demonstrating a higher statistical efficacy (Figure 1). The GLM exhibits limited control over false positive rates, leading to elevated false positive rates in the results of association analyses (Supplementary Figure S2). Consequently, the FarmCPU was identified as the most effective association model for GWAS in this research. The analysis results of this model (Supplementary Table S2) revealed a total of 29 significant SNPs. Among these, fifteen SNPs were associated with HKW, five SNPs were linked to KL, and nine SNPs were related to KW.

### 2.3. Screening and Functional Analysis of Candidate Genes

Based on the localization of this population, the SNP binding linkage disequilibrium (LD) attenuation distance was set to 50 kb (R^2^ = 0.2) [21]. Candidate genes were identified in the B73_RefGen_v4 reference genome, utilizing a 50 kb interval both upstream and downstream of the SNPs as the candidate region. A total of 60 genes were identified in the HKW candidate area, while 19 genes were found in the KL candidate area, and 18 genes were detected in the KW candidate area (Supplementary Table S3).

To assess the tissue-specific expression of the candidate genes, we analyzed their expression characteristics across the primary developmental stages and tissues of grains using qTeller analysis from the MaizeGDB database. This allowed us to identify and screen for genes associated with kernel development. The results indicate that, as shown in Supplementary Table S4, 76 of the 97 genes in the candidate region are expressed in kernel tissue and during various developmental stages.

Some genes exhibit high expression levels at relevant stages and in specific tissues of kernel development (Supplementary Figure S3). Notably, *Zm00001d006871* and *Zm00001d039914* are extremely highly expressed across most stages and tissues. In contrast, *Zm00001d041498*, *Zm00001d044154*, *Zm00001d013175*, *Zm00001d021742*, and *Zm00001d011892* are not expressed in most stages and tissues, with very low expression observed only in select stages and tissues.

Based on gene function annotation, we identified six potential candidate genes, primarily encoding a variety of proteins and transcripts related to growth and development factors (Table 2).

### 2.4. Basic Characteristics of QTLs Related to Maize Kernel Traits

This study compiled 765 QTLs for meta-QTL analysis from 56 QTL localization reports concerning maize 100-kernel weight, kernel width, and kernel length (Supplementary Table S5). Among them, HKW-related QTLs accounted for the largest proportion (414), followed by KW-related QTLs (196), and KL-related QTLs (155) (Supplementary Table S6). They are unevenly distributed across each chromosome, with a maximum of 131 QTLs on chromosome 1 and a minimum of 24 QTLs on chromosome 8 (Supplementary Figure S4A). It is noteworthy that the PVE explained by more than 60% of the initial QTLs was less than 10% regardless of the grain trait (Supplementary Figure S4B), indicating that the three traits of KL, KW and HKW of maize were mainly controlled by micro-effect polygenes and had complex genetic structures.

### 2.5. Meta-QTL Analysis

**Figure 2:**
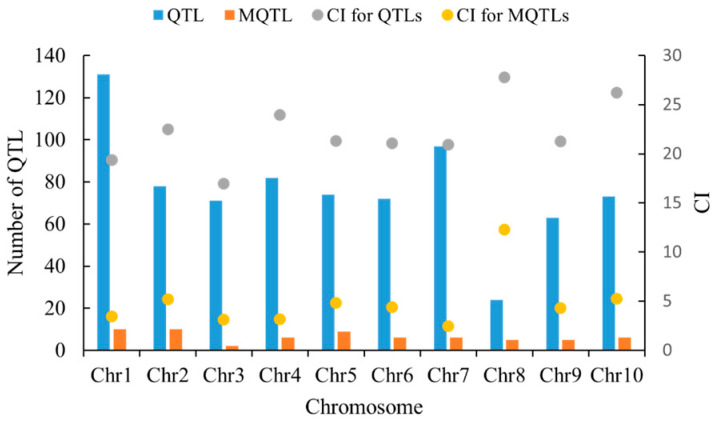
Basic information about MQTL.

In this study, 765 QTLs were projected onto the IBM2 2008 Neighbors reference map by BioMercator v.4.2 software, and a total of 678 QTLs were mapped, accounting for 88.6% of the total QTLs (Supplementary Figure S5). The remaining QTLs did not map to the reference map, which we hypothesize may be due to a lack of common markers between the original QTL and the reference map, or possibly a result of lower PVE and larger CI [22]. Based on the model with the lowest AIC value and the principle that the number of initial QTLs is no less than three, a total of 65 MQTLs associated with grain traits were identified (Supplementary Table S7). These MQTLs were found to be unevenly distributed across 10 corn chromosomes. Among them, chromosomes 1 and 2 had the most MQTLs (ten each), chromosome 5 had nine MQTLs, chromosomes 4, 6, 7, and 10 each had six MQTLs, chromosomes 8 and 9 each had five MQTLs, and chromosome 3 had the least amount of MQTLs (only two). Each MQTL contained 19~126 initial QTLs, and the average confidence interval of a single MQTL was about 4.78 cM, which is nearly 77.84% smaller than that of the initial QTL confidence interval (Figure 2).

**Figure 3:**
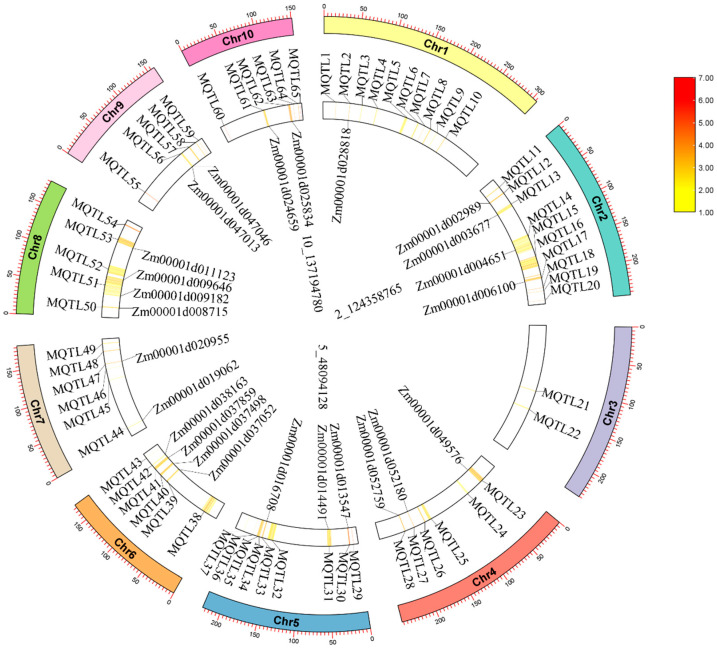
Circos plot of MQTL and significant SNPs distribution in this study. From the inside to the outside, there were three SNPs that were significant in the MQTL interval, the genes related to grain size, the physical mapping position of MQTL, and the chromosome length.

By comparing the location of genetic markers at both ends of the MQTL on the B73 genome (AGPv 4), a total of 5203 candidate genes were identified in these MQTL regions (Supplementary Table S8), with the most candidate genes identified in MQTL 51 (506 in total), and only 2 candidate genes identified in MQTL 36, and MQTL34 fully contained the candidate genes of MQTL35. Twenty-five functional genes and reported candidate genes associated with maize kernel traits were detected in 23 MQTLs (Figure 3). Thus, it is feasible to utilize meta-QTL analysis to investigate genes related to maize kernel traits, and it also indicates that the results of meta-QTL analysis in this study are reliable.

The significant SNPs of 29 kernel traits detected by GWAS were compared with the physical coordinates of 65 MQTLs, and SNPs 5_16083608, SNPs 2_124358765 and SNPs 10_137194780 were located in the MQTL14, MQTL31 and MQTL63 intervals, respectively (Figure 3). In addition, there are 10 significant SNPs that are not in the MQTL interval, but are very close to the MQTL interval (distance < 2 Mb). These results further confirm the accuracy of the significant SNPs associated with kernel traits in this study.

### 2.6. Homologous Gene Mining

We collected 25 genes that were functionally characterized as regulating grain size and weight from the rice genome, and a total of 26 corn homologous genes were found in the MQTL interval through sequence alignment (Supplementary Table S9). *OsAGSW1* has two homologous genes in maize, while the other rice genes have only one homologous gene in maize. These 26 genes are unevenly distributed across 19 MQTL intervals. Among them, the MQTL53 interval contains three genes: *OsCYP20-2* (Gene ID: *Zm00001d011104*), *RL17* (Gene ID: *Zm00001d011353*), and *OsCTPS1* (Gene ID: *Zm00001d011357*), all of which are associated with traits related to grain size and weight. *OsCYP20-2* encodes a thylakoid lumenal cyclophilin that interacts with *OsSYF2* and regulates rice grain length through the splicing of mRNA precursors. In comparison to the wild type, the knockout mutant *oscyp20-2* t1 exhibits shorter grain length, as well as decreased grain width and weight [23]. *RL17* encodes the vacuole protein sorting-related protein OsSNF7.2, which significantly decreases both grain weight and grain width compared to the wild type [24]. *OsCTPS1* may serve as a structural component during endosperm development, participating in microtubule formation through its interaction with tubulin. Overexpression of *OsCTPS1* led to an increase in grain length and width in rice, suggesting that *OsCTPS1* promoted endosperm nucleus separation by participating in microtubule function, affected early endosperm development, and positively regulated rice grain size and weight [25]. Homologs of other grain size and weight-related genes, such as *OsCKX4*, *OsGRX6*, *SG1*, *OsSAPK3*, *qGW8*, etc., were also identified in the MQTL region. These 26 homologous genes were expressed in the main kernel development stages and tissues by qTeller analysis in the MaizeGDB database (Supplementary Table S10), indicating that the homologs of these kernel-related genes may play an important role in maize kernel development.

### 2.7. RT-qPCR Verification

**Figure 4:**
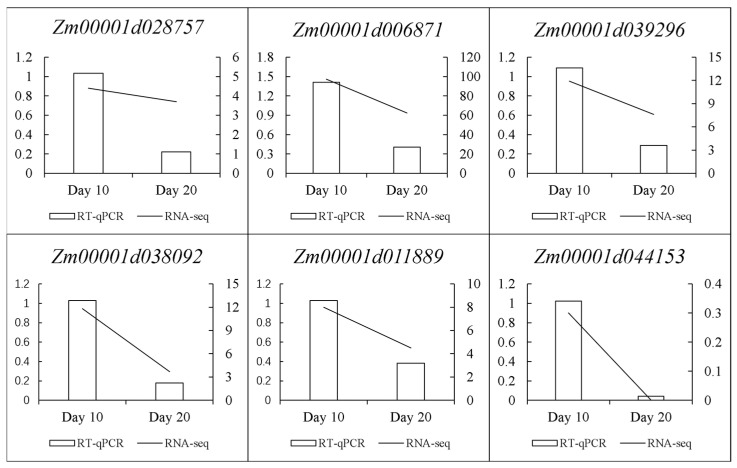
RT-qPCR Verification of six candidate genes.

To assess the reliability of candidate genes, we selected maize kernels at two periods 10 and 20 days after pollination and quantitatively verified the six candidate genes (Figure 4). The results indicate that the RNA-seq data obtained from the MaizeGDB public database are generally consistent with the RT-qPCR findings, thereby demonstrating the reliability of the outcomes of this study.

## 3. Discussion

### 3.1. Genetic Mechanisms of Kernel Size-Related Traits in Maize

Maize kernels are a critical factor that determines yield levels. An in-depth study of the genetic mechanisms underlying maize kernel traits is essential for enhancing yield. Currently, some researchers have made significant progress in investigating the genes associated with maize kernel traits. Zhang et al. [26] identified the gene *ZmKW1*, which regulates maize kernel weight through fine localization and associated localization. Their findings indicate that overexpression of *ZmKW1* impacts endosperm filling by decreasing both the number and size of endosperm cells, ultimately leading to smaller kernels and reduced weight. Zhang et al. [27] conducted a study on miRNA zma-miRNA169o transgenic plants and discovered that miRNA zma-miR169o regulates cell division in maize endosperm. The overexpression of miRNA zma-miR169o significantly promotes cell proliferation in the central endosperm, leading to the production of larger seeds, which in turn increases both kernel size and weight. Consequently, this enhancement results in a significant increase in maize yield. Sun et al. [28] found that the transcription factor ZmBES1/BZR1-5 can forwardly regulate maize kernel size, providing new information for analyzing the development mechanism of maize kernel. Kernel traits are complex characteristics influenced by multiple genes. Further research is necessary to thoroughly analyze the genetic mechanisms underlying maize kernel traits. Therefore, this study predicts six candidate genes related to maize kernel traits, which will provide a theoretical basis for the subsequent analysis of the genetic mechanism of maize kernel traits.

### 3.2. Predictive Analysis of Candidate Gene Function

We identified a total of six candidate genes associated with maize kernel traits. Among these, *Zm00001d028757* encodes the transcription factor bHLH140. BHLH transcription factors are one of the largest family of transcription factors, including two regions with highly conserved basic regions and basic Helix-Loop-Helix (bHLH) [29]. Numerous studies have demonstrated that bHLH transcription factors play a crucial role in the regulation of seed growth and development. Guo et al. [30] ectopically overexpressed the bHLH protein TaPGS1 in wheat and rice, resulting in an increase in grain size and grain weight (up to 13.81% in wheat and 18.55% in rice). Heang et al. [31] discovered that the bHLH protein POSITIVE REGULATOR OF GRAIN LENGTH 1 (PGL1) and PGL2 form heterodimers, inhibiting the function of ANTAGONIST OF PGL1 (APG), affecting cell length, and positively regulating rice grain length. *Zm00001d006871* encodes 40S ribosomal protein SA (RPSA). The ribosomes of eukaryotic cells are composed of a small 40S subunit combined with a large 60S subunit, and ribosomal proteins are important components of ribosomes, mainly involved in RNA processing, DNA repair, and other processes, and play an important role in the regulation of cell proliferation, apoptosis, and development [32]. RPSA is a ribosomal small subunit protein, which is extremely highly expressed in the main process of grain development, except for the 40S small subunit involved in the assembly of ribosomes, and we speculate that it plays an important role in grain development. *Zm00001d039296* encodes Casein Kinase I (CKI). CKI is a highly conserved serine/threonine protein kinase found in various eukaryotic organisms that regulates growth, development, and signal transduction by mediating the phosphorylation of substrates. In Arabidopsis, CK1 protein AELs facilitate the phosphorylation of the transcription factor C3H17, thereby enhancing its protein stability and transcriptional activity, which in turn regulates embryonic development in Arabidopsis [33]. *Zm00001d038092* encodes RING/U-box superfamily protein. As the main E3 ubiquitin ligase, the RING/U-box protein is relatively conserved among various species, mainly regulating plant growth and development, biological stress and abiotic stress [34,35]. In Arabidopsis, the ubiquitin receptor DA1 works synergistically with the E3 ubiquitin ligase DA2 and ENHANCER1 OF DA1 (EOD1)/BIG BROTHER to limit the growth of Arabidopsis seeds [36]. Hu et al. [37] found that the MaU-box gene family of bananas has the highest expression in the early stages of the fruit, and it is speculated that they may play a key role in the growth and development of the fruit. *Zm00001d011889* encodes hexokinase9. Hexokinase is a ubiquitous protein in all organisms and plays an important role in metabolism, glucose signaling, and phosphorylation of glucose and fructose [38]. In rice, hexokinase positively regulates grain size by promoting the development of spikelet shell cells [39]. *Zm00001d044153* encodes cytochrome P450 10. Studies have demonstrated that the cytochrome P450 gene primarily influences plant growth and development by participating in the biosynthesis and catabolism pathways of plant hormones [40]. In rice, the cytochrome P450 subfamily gene *GW10* is involved in the regulation of rice grain size and grain number mediated by rapeseed steroids [41].

### 3.3. Analysis of the Genetic Basis of Maize Kernel Traits

GWAS and meta-QTL analysis represent two effective strategies for dissecting the genetic basis of complex quantitative traits in crops, which have been widely applied across various crop species. The integration of these two approaches enables rapid identification of candidate genes associated with complex quantitative traits in crops [42,43,44]. In the present study, we conducted a meta-analysis of 765 initial QTLs and identified 65 MQTLs. Among these, 25 previously reported functional genes and candidate genes related to maize kernel traits were detected within 23 MQTLs, demonstrating that meta-QTL analysis is an effective and feasible approach for mining genes associated with maize kernel size traits. Through GWAS analysis of three maize kernel traits, we identified 29 significant SNPs, among which three were located within MQTL intervals and ten were in close proximity to MQTL regions (distance < 2 Mb). These findings provide substantial evidence supporting the reliability of the detected SNPs associated with kernel-related traits in our study. The mutually verified SNPs and MQTL intervals will be prioritized for subsequent candidate gene mining related to maize kernel traits. Through comprehensive gene annotation analysis and utilization of RNA-seq data from the MaizeGDB database, we successfully identified and predicted six highly plausible candidate genes. The reliability of these findings was further confirmed through RT-qPCR validation, demonstrating the accuracy and robustness of our research results. Comparative genomic analysis across species serves as a powerful approach for identifying key genes associated with complex quantitative traits in crops [45,46,47]. Utilizing 25 rice genes related to grain traits, we identified 26 homologous genes in maize. Notably, four maize homologs were located within three MQTL intervals validated by significant SNPs, suggesting their potential superiority in regulating maize kernel size and weight compared to other homologs. Within the MQTL31 region, we identified two maize homologs, *Zm00001d014507* and *Zm00001d014447*, corresponding to rice genes *RST1* and *OsSCP46*, which are known to regulate grain size and weight in rice. In rice, *RST1* encodes an auxin response factor (OsARF18) with transcriptional repressor activity, controlling growth and development through auxin signaling [48]. *OsSCP46*, predominantly expressed in the embryo, endosperm, and aleurone layer, negatively impacts grain size, length, width, and thousand-grain weight when knocked out [49]. The MQTL48 region harbors a maize homolog (*Zm00001d021701*) of *OsbHLH57*, which encodes a basic Helix-Loop-Helix (bHLH) transcription factor. Overexpression of *OsbHLH57* enhances grain yield by increasing seed setting rate and grain size under both normal and low-temperature conditions, while its disruption leads to reduced grain dimensions and yield [50]. In the MQTL58 region, we identified *Zm00001d048008*, a maize homolog of *TUD1*, which encodes a U-box E3 ubiquitin ligase involved in brassinosteroid (BR) signaling. *TUD1* interacts with the heterotrimeric G protein α-subunit D1 to regulate BR-mediated rice growth [51]. Furthermore, other homologous genes within MQTL regions have been demonstrated to regulate grain size and weight in rice, suggesting their potential conserved functions in maize. Collectively, these genes may directly or indirectly participate in the regulation of maize kernel size and weight, ultimately influencing yield potential. Successful cloning and functional validation of these candidate genes in future studies would significantly enhance our understanding of the genetic mechanisms underlying maize kernel traits and provide reliable technical support for marker-assisted selection breeding in maize.

## 4. Materials and Methods

### 4.1. Material Planting, Experimental Design, and Phenotypic Trait Determination

The experimental materials used in this study were sourced from the CUBIC population, cultivated by Huazhong Agricultural University. This population is a multi-parent hybrid population with different plant morphology and seed size. These materials were provided by the maize research team at the Agronomy College of Gansu Agricultural University. The planting occurred in 2021 and 2022 at the Huangyang Testing Site of the Gansu Academy of Agricultural Sciences (Qilian Mountain alluvial plain, 1720 m above sea level). In 2023, the materials were planted at the Heheng Maize Research Institute’s planting base in Lanzhou, Gansu Province, China (mountain, 1590 m above sea level).

The fields in both locations were designed randomly according to an experimental design, with one repetition, row length of 2 m, row spacing of 0.5 m and a plant spacing of 0.2 m, forming a single-row area. A total of 10 plants were planted in each row, and the management practices employed were consistent with conventional field management. After the experimental materials are mature, starting from the third plant in each row, three solid and plump corn ears with consistent growth are picked for drying, and the dried ears are mixed and threshed. After removing impurities and abnormal kernels such as mold, corrupt, extremely large, and extremely small kernels, they were randomly selected from them for investigation of maize kernel traits. The traits examined include kernel length (unit: mm), kernel width (unit: mm) and 100-kernel weight (unit: g). The measurement of maize kernel traits was carried out by the maize seed examination and analysis system (model number: TPKZ-1-G) provided by Zhejiang topu Yunnong Technology Co., Ltd., Hangzhou, China. It can automatically detect and analyze the particle type parameters of single-grain corn seeds, including length, width, aspect ratio, perimeter, and area, through image recognition technology [52]. Additionally, it measures the total number of seeds, thousand kernel weight, and hundred kernel weight. Each material underwent three repeated measurements, with a random selection of 150 kernels for analysis.

### 4.2. Phenotypic Data Analysis

Microsoft Excel 2010 was utilized to systematically organize the experimental data. IBM SPSS Statistics 26 (version number: 26.0.0) was employed to conduct descriptive statistical analysis and correlation analysis of the phenotypic data. Additionally, the R programming language [53] was used to analyze the frequency of the phenotypic data and to generate the corresponding charts.

### 4.3. GWAS

In this study, the GAPIT [54] package in R language was used to analyze the kernel correlation traits such as kernel length, kernel width and 100-kernel weight of 1283 maize inbred materials in CUBIC population. Genotype data were obtained from the NCBI website (https://www.ncbi.nlm.nih.gov/bioproject/PRJNA597703, accessed on 1 November 2024). The correlation analysis between traits and SNP markers was conducted using generalized linear model (GLM) and fixed and random model circulating probability unification (FarmCPU) approaches. The previous generation assessed the degree of attenuation of linkage disequilibrium (LD) within this associated group using 11.8 M high-quality SNP markers. The results indicate that the optimal LD attenuation distance for this group is 50 kb (R^2^ = 0.2). GWAS results are presented in R language to draw Manhattan graphs and QQ graphs.

### 4.4. Collection of Initial QTL Information

We utilized Web of Science (http://www.webofknowledge.com, accessed on 1 November 2024), PubMed (https://www.ncbi.nlm.nih.gov/pubmed, accessed on 1 November 2024), and CNKI (https://www.cnki.net, accessed on 1 November 2024) to search for keywords such as maize, kernel size, QTL, kernel length, kernel width, 100-kernel weight, and yield in order to gather information on QTL related to maize kernel traits reported over the past 20 years. The collected QTL information includes population type, population size, mapping method, chromosome location, LOD value, phenotypic variance explained (PVE), confidence interval, genetic map marker, etc. If the LOD value is absent, it is assumed to be 3; If the PVE is not provided, the QTL is excluded. If the confidence interval (CI) is not provided, the following formula is employed to calculate it based on the type of population:(1) CI = 287/(population size × PVE), applied to double-haploid (DH) population; (2) CI = 163/(population size × PVE), applied to recombinant inbred line (RIL) population; (3) CI = 530/(population size × PVE), applied to F_2_ and backcross (BC) population [55,56,57].

To initiate the meta-QTL analysis, download the IBM2 2008 Neighbors high-density molecular linkage map from the MaizeGDB website (https://www.maizegdb.org/data_center/map, accessed on 1 November 2024) to serve as the reference map for integrating QTL from various sources. Next, obtain the BioMercator v.4.2 software from the following link: https://urgi.versailles.inra.fr/Tools/BioMercator-V4 (accessed on 1 November 2024). After acquiring the software, configure the necessary genetic map and QTL files according to the software’s specifications. Finally, import the sorted QTL files and genetic map files into BioMercator v.4.2 to commence the meta-QTL analysis.

Using the Akaike information criterion (AIC), AIC correction (AICc), AIC 3 candidate models (AIC3s), Bayesian information criterion (BIC), and average weight of evidence (AWE) derived from the simulation operation, all possible QTL combinations are evaluated. The optimal model is determined based on the minimum AIC value. Furthermore, the criteria for meta-QTL selection stipulate that there must be no fewer than three initial QTLs [58].

### 4.5. Candidate Gene Mining and Functional Analysis

For the results of the GWAS analysis, since the linkage imbalance attenuation distance of this population is 50 kb (R^2^ = 0.2), the physical location of the SNPs marked significantly related to the target trait is 50 kb, i.e., in the range of 100 kb. MaizeGDB (https://maizegdb.org/genome/assembly/Zm-B73-REFERENCE-GRAMENE-4.0, accessed on 1 December 2024) was used to search all candidate genes marked with significantly related SNPs in the target trait and select genes as the best candidate gene based on gene function annotation. RNA_seq data of tissue sites related to grain development of the B73 inbred line were downloaded from MaizeGDB, and tissue-specific expression analysis of candidate genes was performed. For the results of the meta-QTL analysis, two flanking marks of MQTL can be obtained based on the location of MQTL and CI (95%). Then, the physics of some flanking marks can be found on the IBM2 2008 Neighbors high-density molecular linkage map in the MaizeGDB database. Location, and then use MaizeGDB (https://maizegdb.org/genome/assembly/Zm-B73-REFERENCE-GRAMENE-4.0, accessed on 1 December 2024) to find candidate genes contained in the MQTL interval.

### 4.6. Mining of Homologous Genes

In this study, we collected 25 genes associated with rice grain traits from the China Rice Data Center (https://www.ricedata.cn, accessed on 1 December 2024) to identify homologous genes in maize. The protein sequences of these 25 rice genes were retrieved from the same website. These sequences were subsequently utilized to conduct BLASTP searches against the maize non-redundant protein sequence database (https://www.ncbi.nlm.nih.gov, accessed on 1 December 2024). The identification of homologous genes was performed using stringent screening criteria, including an E-value threshold of <1 × 10^−10^, query coverage exceeding 60%, sequence identity greater than 60%, and a minimum alignment score of 200.

### 4.7. Real-Time Fluorescence Quantitative PCR (RT-qPCR) Verification of Candidate Genes

The experimental materials consisted of the maize B73 inbred line, supplied by the maize research team at Gansu Agricultural University. These materials were planted in 2024 at the Heheng Maize Research Institute’s planting base in Lanzhou, Gansu Province, China (mountain, altitude of 1590 m). After pollination, kernels from the middle of the maize ears were harvested on the 10th and 20th days post-pollination for RNA extraction from the whole seeds. RNA extraction was performed using the RNAprep Pure Plant Total RNA Extraction Kit provided by TIAN GEN. Reverse transcription and real-time fluorescence quantification were conducted using Accurate Biotechnology’s Evo M-MLV reverse transcription reagent and the SYBR Green Pro Taq HS premixed qPCR kit (with ROX), utilizing actin as the internal reference gene. Specific primers for candidate genes were designed using NCBI, and RT-qPCR was conducted on a QuantStudio 5 real-time PCR system. The relative expression levels of the candidate genes were calculated using the 2^−ΔΔCt^ method [59], and the subsequent results were analyzed.

## 5. Conclusions

In this study, we identified 29 significantly associated SNPs through GWAS analysis of three kernel-related traits, predicting six candidate genes potentially associated with kernel size and weight characteristics. Furthermore, our meta-QTL analysis of kernel-related traits identified 65 MQTLs, among which 25 previously reported functional genes and candidate genes related to maize kernel traits were detected within 23 MQTLs. Additionally, we identified 26 maize homologous genes through comparative analysis of 25 rice genes associated with grain size and weight. These candidate genes provide novel insights into the genetic mechanisms underlying maize kernel size. The findings of this study will provide theoretical support for the map-based cloning of genes related to maize kernels and molecular marker-assisted breeding selection.

## Figures and Tables

**Table 1:** Phenotypic identification of maize grain traits.

Trait	Mean	Range	CV/%	Skewness	Kurtosis
KL	9.50 ± 0.78	7.05–13.1	8	0.26	0.53
KW	7.90 ± 0.66	5.58–10.81	8	0.1	0.3
HKW	26.62 ± 4.86	9.84–53.78	18	0.33	0.88

Note: KL, KW and HKW respectively represent kernel length, kernel width and 100-kernel weight. CV stands for coefficient of variation.

**Table 2:** Candidate gene functional annotation.

Trait	SNPs	Gene	Annotation
HKW	1_45747417	*Zm00001d028757*	transcription factor bHLH140
HKW	2_218329593	*Zm00001d006871*	40S ribosomal protein SA-1
HKW	3_1222238	*Zm00001d039296*	Casein Kinase I
HKW	6_147537023	*Zm00001d038092*	RING/U-box superfamily protein
HKW	8_164076212	*Zm00001d011889*	hex9—hexokinase9
KW	3_220626790	*Zm00001d044153*	cyp10—cytochrome P450 10

Note: HKW and KW respectively represent 100-kernel weight and kernel width.

## Data Availability

Data are contained within the article and the Supplementary Materials.

## Supplementary Materials

The following supporting information can be downloaded at https://www.mdpi.com/article/10.3390/plants14060959/s1: Figure S1: Frequency histogram of hundred kernel weight, kernel length and kernel width of maize; Figure S2: Manhattan and QQ plots of maize grain-related traits under two GWAS model analyses; Figure S3: Expression calorigrams of candidate interval genes in the main developmental stages and tissues of maize grains; Figure S4: Basic characteristics of QTLs associated with maize grain traits; Figure S5: Projection and distribution of QTLs and MQTLs (Meta QTLs) identified based on grain traits on chromosome 1; Table S1: Correlation analysis of maize grain traits; Table S2: Significant SNPs of maize kernel traits; Table S3: Candidate genes within the SNP interval associated with maize kernel traits; Table S4: Expression data of candidate region genes in grain organization and developmental stages; Table S5: Collection of initial QTL; Table S6: Information for the initial QTL; Table S7: Description of MQTLs in this study; Table S8: Genes in MQTL intervals; Table S9: Maize homologous genes in rice; Table S10: Expression data of homologous genes in maize grain organization and developmental stages. References [60,61,62,63,64,65,66,67,68,69,70,71,72,73,74,75,76,77,78,79,80,81,82,83,84,85,86,87,88,89,90,91,92,93,94,95,96,97,98,99,100,101,102,103,104,105,106,107,108,109,110,111,112,113,114,115,116,117,118,119,120,121,122,123,124,125,126,127,128,129,130,131,132,133,134,135,136,137,138,139,140,141,142,143,144,145,146,147,148] are cited in the supplementary materials.
