# Supplementary material for: Candidate Gene for Kernel-Related Traits in Maize Revealed by a Combination of GWAS and Meta-QTL Analyses
Source: Plants (Basel). 2025 Mar 19;14(6):959. doi: 10.3390/plants14060959 (PMC11946461; doi:10.3390/plants14060959)
Supplement: Supplementary file 1 [file plants-14-00959-s001.zip › Supplemental Figure.pdf]

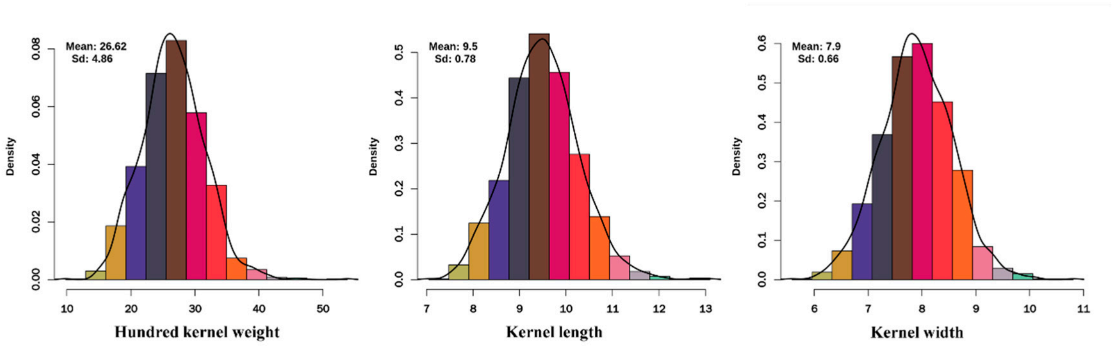

Supplementary Figure S1. Frequency histogram of hundred kernel weight, kernel length and kernel width of maize.

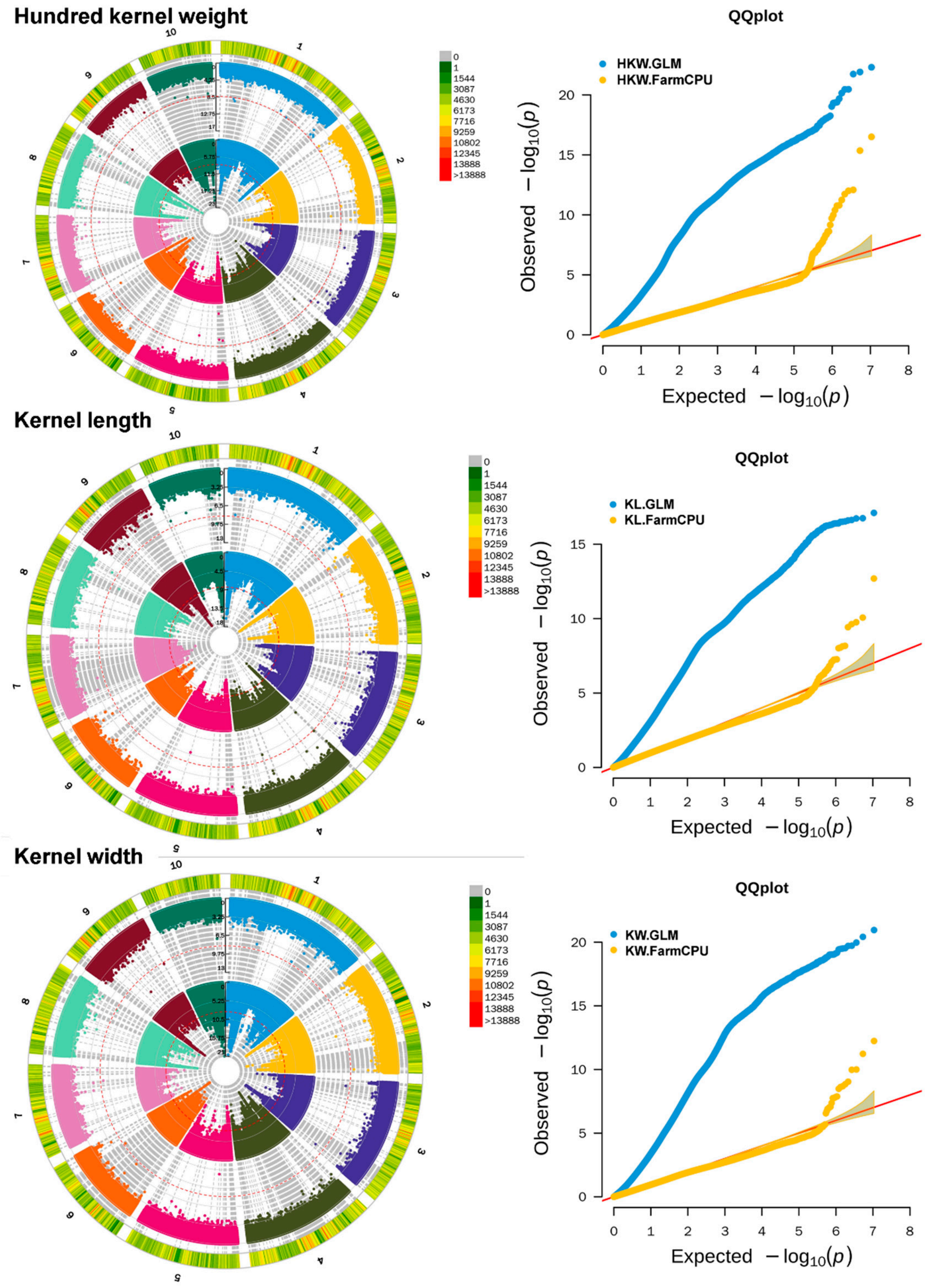

Supplementary Figure S2. Manhattan and QQ plots of maize grain-related traits under two GWAS model analyses. The Manhattan diagram is the results of the FarmCPU model and the GLM model from the outside to the inside. HKW, hundred kernel weight; KL, Kernel length; KW, Kernel width. The red dotted line represents the threshold line.

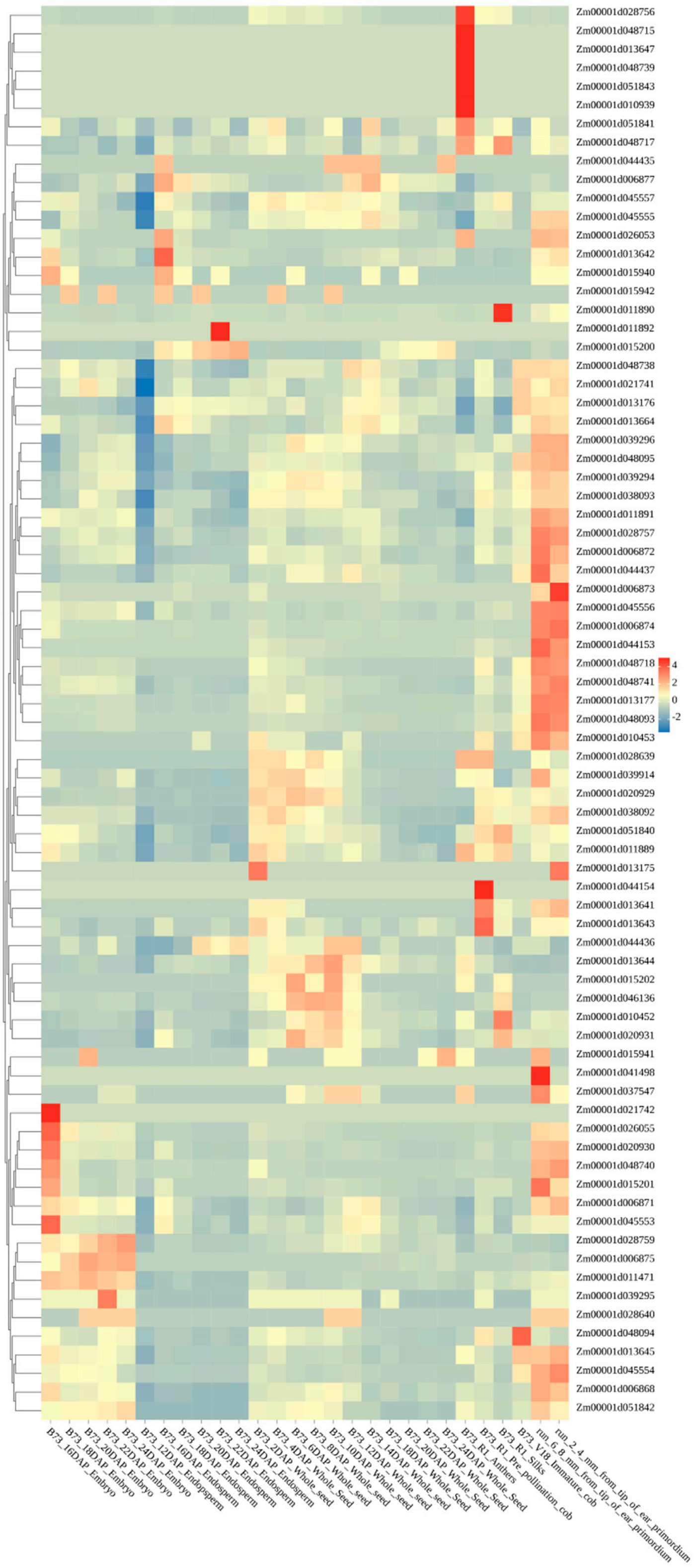

Supplementary Figure S3. Expression calorigrams of candidate interval genes in the main developmental stages and tissues of maize grains.

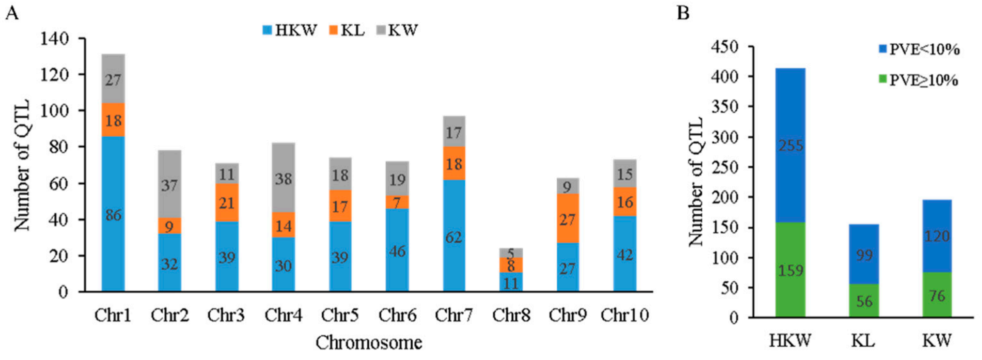

Supplementary Figure S4. Basic characteristics of QTLs associated with maize grain traits. (A) Genomic distribution of QTLs associated with maize grain traits; (B) PVE characteristics of the initial QTL.

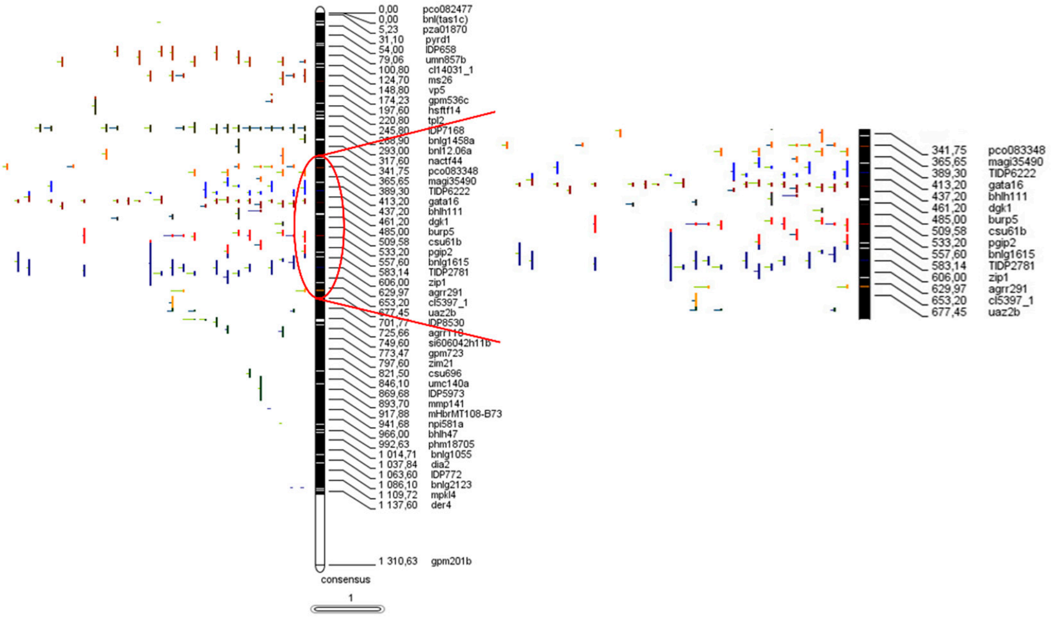

Supplementary Figure S5. Projection and distribution of QTLs and MQTLs (Meta QTLs) identified based on grain traits on chromosome 1. The bars on the left side of the chromosome correspond to the QTLs associated with the grain trait, the black bars on the chromosome represent the marker density, the colored fragments on the chromosome represent the MQTLs, and the right side of the chromosome is the molecular marker and genetic distance (cM).
